# Effectiveness of Photodynamic Therapy and Nd-YAG Laser Treatment for Obstructed Tracheobronchial Malignancies

**DOI:** 10.1155/DTE.5.161

**Published:** 1999

**Authors:** Kinya Furukawa, Tetsuya Okunaka, Hideki Yamamoto, Takaaki Tsuchida, Jitsuo Usuda, Hideo Kumasaka, Junzou Ishida, Chimori Konaka, Harubumi Kato

**Affiliations:** Department of Surgery Tokyo Medical University 6-7-1 Nishishinjuku Shinjuku-ku Tokyo 160-0023 Japan

## Abstract

Since 1980, advanced lung carcinomas were treated with palliative laser therapy for the purpose of opening the endobronchial stenosis and obstruction by either photodynamic therapy (PDT) or Nd-YAG laser treatment at Tokyo Medical University. A total of 258 lesions were treated, 81 by PDT and 177 by Nd-YAG laser treatment. PDT achieved effective results in 61 (75%) of 81 lesions. In the Nd-YAG laser group, 143 (81%) of 177 lesions showed effective results. When the tumor was located in the trachea or main bronchi, effective results were obtained in 73% (19 of 26) of cases treated by PDT and in 93% of cases (64 of 69) treated by Nd-YAG laser. However, in cases in which the tumor was located in lobar or segmental bronchi, the tumor response was effective in 76% (42 of 55) of PDT-treated patients and 73% (79 of 108) of Nd-YAG laser-treated patients. With a mortality rate of 0%, the greatest advantage of PDT over Nd-YAG treatment was safety. Considering complications, PDT seems to be useful for obstruction of lobar and segmental bronchus. Nevertheless, when deciding among alternative therapies, physicians treating patients with advanced lung carcinoma should give careful consideration to the benefit and complications of both laser therapies and decide the most suitable modality.


*Diagnostic and Therapeutic Endoscopy*, Vol. 5, pp. 161–166
Reprints available directly from the publisher
Photocopying permitted by license only

© 1999 OPA (Overseas Publishers Association) N.V.
Published by license under
the Harwood Academic Publishers imprint,
part of The Gordon and Breach Publishing Group.
Printed in Malaysia.

### Effectiveness of Photodynamic Therapy and Nd-YAG Laser Treatment for Obstructed Tracheobronchial Malignancies

KINYA FURUKAWA*, TETSUYA OKUNAKA, HIDEKI YAMAMOTO, TAKAAKI TSUCHIDA, JITSUO USUDA, HIDEO KUMASAKA, JUNZOU ISHIDA, CHIMORI KONAKA and HARUBUMI KATO

*Department of Surgery, Tokyo Medical University, 6-7-1 Nishishinjuku, Shinjuku-ku, Tokyo 160-0023, Japan*

**Since 1980, advanced lung carcinomas were treated with palliative laser therapy for the purpose of opening the endobronchial stenosis and obstruction by either photodynamic therapy (PDT) or Nd-YAG laser treatment at Tokyo Medical University. A total of 258 lesions were treated, 81 by PDT and 177 by Nd-YAG laser treatment. PDT achieved effective results in 61 (75%) of 81 lesions. In the Nd-YAG laser group, 143 (81%) of 177 lesions showed effective results. When the tumor was located in the trachea or main bronchi, effective results were obtained in 73% (19 of 26) of cases treated by PDT and in 93% of cases (64 of 69) treated by Nd-YAG laser. However, in cases in which the tumor was located in lobar or segmental bronchi, the tumor response was effective in 76% (42 of 55) of PDT-treated patients and 73% (79 of 108) of Nd-YAG laser-treated patients. With a mortality rate of 0%, the greatest advantage of PDT over Nd-YAG treatment was safety. Considering complications, PDT seems to be useful for obstruction of lobar and segmental bronchus. Nevertheless, when deciding among alternative therapies, physicians treating patients with advanced lung carcinoma should give careful consideration to the benefit and complications of both laser therapies and decide the most suitable modality.**

*Keywords:* Endobronchial tumor, Nd-YAG laser, Photodynamic therapy

### INTRODUCTION

Endoscopic surgery using laser is recognized as an effective modality for endobronchial carcinoma. Recently, however, attention has increasingly been focused on photodynamic therapy (PDT) with photosensitizer, i.e., Photofrin®. The Japanese government approved the use of this modality for early stage lung carcinoma in October 1995, and reimbursement through the National Health Insurance began in April 1996. The result of PDT for early stage lung carcinoma at our institution

* Corresponding author. Tel.: 81-3-3342-6111, ext. 5071. Fax: 81-3-3349-0326.

showed an excellent complete remission rate (83.7%). Therefore, PDT is a good indication for central type early stage lung carcinoma.

However, little can be done in cases of advanced stage lung carcinoma. Only 12% of stage III patients survive for 2 years regardless of therapy [1,2]. Transbronchial resection using the neodymium : yttrium–aluminum–garnet (Nd-YAG) laser has become well accepted after the initial reports of its efficacy were published. Transmitted through optical fibers, the Nd-YAG laser provides excellent vaporization, low absorption by hemoglobin, and good tissue penetration 5–10 mm from the focal point with high-energy output. Good results were reported in cases of symptomatic endobronchial tumor obstruction treated with the Nd-YAG laser [3]. The drawback of this laser, however, is its nonspecificity. The Nd-YAG laser produces a beam that not only vaporizes or coagulates tumor tissue but that may also harm normal bronchial tissues and thus lead to severe bleeding or perforation of the bronchial wall. Treatment with the Nd-YAG laser may improve ventilation of patients with endobronchial tumors, but only partial removal of the tumor should be attempted using this method [4]. With PDT, the endobronchial tumor, if small enough, can be eliminated completely with no fear of complications. In patients in the United States and Europe with lung carcinoma or endobronchial metastatic lesions, the primary use of PDT is for palliation of endobronchial obstruction in patients who have intrinsic lesions of the bronchus that cause partial or complete obstruction [5]. In this paper, we have discussed the effectiveness of PDT and Nd-YAG laser treatments performed at our institution for obstructed lung carcinoma.

### PATIENTS AND METHODS

### Patients

Since 1980, a total of 258 lesions were performed through palliative laser therapy for the purpose of opening the endobronchial stenosis and obstruction by either PDT or Nd-YAG laser therapy at Tokyo Medical University. Among them, PDT group was 31% (81/258), and Nd-YAG laser group was 69% (177/258). The population of PDT group consisted of 72 male and 6 female in a total of 78 cases (81 lesions). The mean age was 65.0 years (ranging from 41 to 84 years). According to the histology, 65 lesions were those of squamous cell carcinoma, 9 lesions in adenocarcinoma, 4 in small cell carcinoma, large cell carcinoma in 2 cases and 1 in metastatic lung tumor. The majority of histology was found in squamous cell carcinoma, which occupied 80% of PDT group.

### Treatment Methods

Both laser treatments were performed by flexible bronchofiberscopy under local anesthesia using 4% xylocaine. Nd-YAG laser system (Olympus, model MYL-2) was used for laser vaporization. The wavelength at 1,064 nm was irradiated to the lesions and the energy was from 20 to 40 W. Non-contact fiber was used and laser irradiation time was 1.0 to 2.0 s repeatedly.

Tumor selective photosensitizer was necessary to perform PDT. As a photosensitizer, hematoporphyrin derivative (HpD; Photofrin Medical Inc., Cheektogawa, NY) or Photofrin® (QOL Inc., Vancouver, Canada) was used for PDT. The photosensitizer was administered at 2.0 mg/kg intravenously and at 48 or 72 h after administration, laser irradiation at 630 nm which is the longest absorption band of HpD and Photofrin® was performed. Since 1980–1985, argon dye laser system (Spectra Physics), and thereafter excimer dye laser system developed by cooperation of Tokyo Medical University and Hamamatsu Photonics Inc. were employed for PDT. The laser beam was transmitted via a 400 nm-quartz fiber inserted in the instrumentation channel of a fiberoptic bronchoscope. The tip of the quartz fiber was maintained at a distance of 1–2 cm from the lesion for surface irradiation or the fiber was inserted into the obstructed tumor (interstitial irradiation). Depends on tumor size, combinations of both irradiation methods were used. Total irradiation energy was 200 J in average.

**Evaluation of Treatment Effects**

All cases were evaluated one month later, except emergent cases for Nd-YAG laser treatment. Cases in which tumor size or the degree of bronchial obstruction was reduced by more than 50% were classified as effective in terms of tumor response. Ineffective cases were those in which tumor size or the degree of obstruction was reduced by less than 50% [5]. Emergent cases performed Nd-YAG laser for the purpose of immediate relief from severe dyspnea were also classified as effective if the tumor was not reduced by more than 50%. We investigated the number of bronchoscopic application and complications to compare the benefits of the both modalities. Furthermore, we also compared the data of $PaO_2$ and performance status (PS) before and after PDT to estimate PDT efficacy.

### RESULTS

A total of 258 lesions were treated, 81 by PDT and 177 by Nd-YAG laser treatment. PDT achieved effective results in 61 (75%) of 81 lesions. In the Nd-YAG laser group, 143 (81%) of 177 showed effective results. Bronchoscopical findings of PDT and Nd-YAG laser before and after treatment were shown in Figs. 1 and 2. In this study, effective tumor response depending on tumor location was also examined. Location of the lesions and the efficacy of laser treatment were shown in Table I. Four lesions (5%) in trachea, 22 lesions (27%) in main bronchus, 36 lesions (44%) in lobar bronchus and 19 lesions (24%) in segmental bronchus were treated by PDT. On the other hand, 69 lesions (39%) in trachea and main bronchus and 108 lesions (61%) in lobar and segmental bronchus were treated by Nd-YAG laser. When the tumor was located in the trachea or main bronchi, effective results were obtained in 73% (19 of 26) of cases treated by PDT and in 93% (64 of 69) treated by Nd-YAG laser. However, in cases in which the tumor was located in lobar or segmental bronchi, the tumor response was effective in 76% (42 of 55) of PDT-treated patients and 73% (79 of 108) of Nd-YAG laser-treated patients.

Complications of PDT and Nd-YAG laser therapy were shown in Table II. No fatal complications occurred in any of the PDT treated patients, whereas 91% (71 of 81) of patients developed slight skin photosensitivity, although no patient required treatment. Other complications of PDT were pneumonia in 4 cases (6%), slight fever in 2 cases

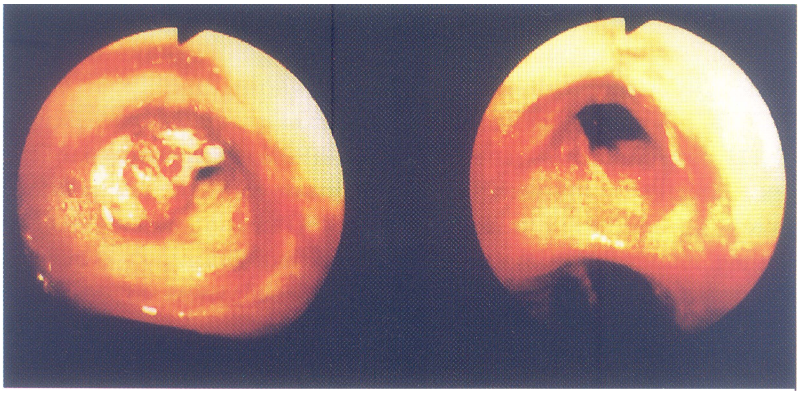

Pre-Treatment Post-Treatment

FIGURE 1 PDT using Photofrin® and excimer dye laser was performed for 90% obstruction of left upper bronchus. Excimer dye laser irradiation was performed at 48 h after 2.0 mg/kg of Photofrin® intravenous injection. The laser energy was adjusted to 4 mJ/pulse with 30 Hz of frequency and the total laser energy was 400 J. Opening of bronchial obstruction was obtained at 2 days after PDT by bronchial toileting.

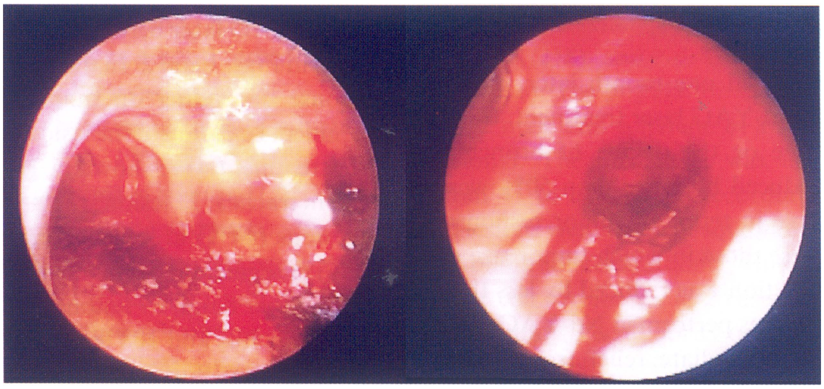

FIGURE 2 Nd-YAG laser vaporization was performed for the complete obstruction of right main bronchus. Total laser energy of Nd-YAG laser was 12,500 J at 30 W. Severe dyspnea was improved dramatically after opening the bronchial lumen.

TABLE I Location of lesions and efficacy of laser treatment

| Location | PDT | Nd-YAG |
|---|---|---|
| Trachea and main bronchus | 73% (19/26) | 93% (64/69) |
| Lobar and segmenta bronchus | 76% (42/55) | 73% (79/108) |
| Total | 75% (61/81) | 81% (143/177) |

TABLE II Complications of laser treatment

| Treatment | Complication | | Mortality |
|---|---|---|---|
| PDT | Photosensitivity | 71 (91%) | |
| | Pneumonia | 4 (6%) | |
| | Slight fever | 2 (3%) | |
| | | | 0 (0%) |
| Nd-YAG | Pneumonia | 12 (7%) | |
| | Massive bleeding | 10 (6%) | |
| | Arrhythmia | 8 (5%) | |
| | Perforation | 4 (3%) | |
| | | | 3 (1.7%) |

(3%), which were not directly related with PDT procedure, but the percentage were very low. Severe complications, including massive bleeding in 10 patients (6%) and bronchial perforation in 4 cases (3%) occurred in the Nd-YAG treated group. As a result of these complications, 3 patients (1.3%) died. The mortality of laser therapy was 0% in PDT, 1.7% in Nd-YAG treatment.

The numbers of bronchoscopic application performed for laser treatment and bronchial toileting were different between the 2 groups (Table III). In the PDT group, laser irradiation was usually only once (91%), but bronchial toiletings after PDT were necessary more than twice (95%). On the contrary, laser irradiation of Nd-YAG laser was necessary more than twice (69%), but less than once (90%) bronchoscopy was sufficient for toileting.

TABLE III Numbers of bronchoscopic application

| Treatment | No. of Tx. | % of cases | % of toileting |
|---|---|---|---|
| PDT | 0 | — | 0 |
| | 1 | 91 | 5 |
| | >2 | 9 | 95 |
| Nd-YAG | 0 | — | 35 |
| | 1 | 31 | 55 |
| | >2 | 69 | 10 |

TABLE IV Evaluation of improvement by PDT

| | Pre-treatment | Post-treatment |
|---|---|---|
| $PaO_2$ (mmHg) | $66 \pm 21$ | $82 \pm 14$ |
| Performance status | $1.8 \pm 0.5$ | $0.8 \pm 0.4$ |

To evaluate quality of life (QOL) of the patients after PDT, we compared the data of $PaO_2$ and performance status (PS) before and after PDT to estimate PDT efficacy in 15 cases (Table IV). The averages of $PaO_2$ and PS were 66 mmHg and 1.8. After PDT, the averages increased to 82 mmHg and 0.8 respectively.

### DISCUSSION

Endoscopic surgery using Nd-YAG laser and PDT has now achieved a status as effective therapeutic modality for endobronchial carcinoma. Especially increasing attention has been focused on PDT using Photofrin® and other photosensitizers. PDT for early stage lung carcinoma obtained government approval in October 1994 and finally obtained Japanese National Health Insurance reimbursement status in April 1996.

Since 1980, 248 patients (296 lesions) with central type lung carcinomas included early stage and advanced carcinoma have been treated in our institute. Overall complete remission was obtained in 42.5% of the 125 lesions, partial remission in 56.8% and no remission was obtained in 1.0%. Especially in early stage lung carcinoma as a curative purpose, CR was obtained in 107 (84.9%) out of 126 cases and 61 cases were disease free at 2 to 200 months. So far in Japan, PDT is approved only for early stage lung carcinoma because of the excellent result. However, PDT has good indications for not only early stage lung carcinoma, but also preoperative laser irradiation for the purposes of increasing operability and reducing the extent of resection area and opening the bronchi for advanced lesions.

In this paper, we have demonstrated the efficacy of PDT and Nd-YAG laser treatment for obstructed lung carcinoma. Overall effective opening of bronchi was achieved in 61 out of 81 lesions (75%) for the PDT treated group, as opposed to 143 of 177 (81%) for the Nd-YAG laser-treated group. When the tumor was located in the trachea or main bronchi, effective results were obtained in 73% (19 of 26) of cases treated by PDT and in 93% (64 of 69) treated by Nd-YAG laser. However, in cases in which the tumor was located in lobar or segmental bronchi, the tumor response was effective in 76% (42 of 55) of PDT treated patients and 73% (79 of 108) of Nd-YAG laser-treated patients. Therefore, Nd-YAG laser treatment was useful for obstruction of large bronchus (trachea and main bronchus), on the other hand, the similar results were obtained by PDT for obstruction of lobar and segmental bronchus.

The procedures also differed in that the laser used in PDT could be inserted safely into the tumor for treatment without perforation or harm to adjacent vessels. With the Nd-YAG laser, however, bronchial wall perforation or massive hemorrhaging resulting from the blood vessel injury sometimes occurred [6]. This study shows that in the bronchial wall, tumor necrosis is not induced as effectively by Nd-YAG laser treatment as it is by PDT. This is true, especially in the smaller bronchi, because of the lesser margin for error with the Nd-YAG laser before entering the vessel. Nd-YAG treatment in distal bronchi is extremely difficult and dangerous. Hemorrhage from pulmonary vessels was one of the major complications of using the Nd-YAG laser, but hemorrhaging did not occur with PDT. Similar to our experience, McCaughan *et al.* had reported no intraoperative death or cases of intraoperative bleeding or smoke with 118 PDT cases [7]. With a mortality rate of 0%, the greatest advantage of PDT over Nd-YAG treatment is safety. Considering the complication, PDT seems to be useful for obstruction of lobar and segmental bronchus.

Toilet bronchoscopy after laser therapy was different in the PDT treated group and the Nd-YAG treated group. Clean up after PDT involved the simple removal of gelatinous or fibrinous plugs from the bronchus in large pieces, but frequent bronchoscopy is stressful for some patients. On the other hand, Nd-YAG treatment required time-consuming piecemeal forceps removal of charred and coagulated tissue resulting from the treatment, but in most cases, the toileting bronchoscopy was sufficient for once.

Since Balchum and Doiron had demonstrated the good result, objective response rate; 98.6% (71/72) of PDT for obstructed endobronchial carcinoma, many investigators has studied the utility of PDT for advanced lung carcinoma [5]. McCaughan *et al.* had demonstrated the effects on arterial blood gas levels after PDT (31 cases) and Nd-YAG laser (86 cases) treatment for advanced endobronchial malignancies [8]. A strong, inverse linear relationships was found in $PaO_2$ and its initial level and between $PaCO_2$ and pH changes. They concluded that PDT is no more harmful for acid-base metabolism and respiratory functions than Nd-YAG laser tumor ablation. Our data also showed the improvement of $PaO_2$ after PDT. Lam *et al.* analyzed the response of 24 patients with obstructed bronchial tumors to PDT [9]. PDT was found to be most effective when the tumor was polipoid appearance, with little or no submucosal invasion or peribronchial extension. Patients who had 50% or more of the endobronchial obstruction due to mucosal tumor had no evidence of local tumor recurrence for a median interval of 22 weeks after PDT. The advantages and disadvantages of PDT compared with the Nd-YAG laser treatment were summarized by McCaughan *et al.* [3]. The disadvantages of PDT are: (a) photosensitizer injection, from which skin photosensitivity may result, (b) the required waiting period between injection and treatment, and (c) the need to perform frequent toilet bronchoscopy. The advantages of PDT treatment are three fold: (a) the technical ease allowed by its safety, thus allowing little chance of perforation and little risk of intraoperative hemorrhaging, (b) no endobronchial smoke, and (c) freedom to insert the fiber blindly into tissue.

Although the advantages of PDT for treatment of advanced obstructing bronchial malignancy are emphasized, the first choice for patients with severe respiratory distress is immediate Nd-YAG laser therapy, because PDT requires a 2 to 3 day waiting period for selective retention of photosensitizer before laser irradiation. After laser irradiation, photodynamic reaction also may cause severe obstruction of bronchi because of mucosal edema and necrotic tissue. Furthermore, patients treated with PDT may be ambulatory, but because they are instructed to avoid direct sunlight, their activity is severely limited for at least 2 weeks of their short remaining life span. Sutedja *et al.* had reported a pilot study of PDT for 26 cases of inoperable non-small cell lung carcinoma, and concluded that the clinical benefit of PDT in advanced cases was small [10]. In these days, second generation photosensitizers for example Npe6 and m-THPC which are able to excite by longer wavelength than Photofrin® has been developed [11,12], so deeper tissue penetration and photodynamic effect are expectable. A randomized trial seems to be necessary to determine whether PDT or Nd-YAG laser treatment is more effective in relieving endobronchial obstruction. Nevertheless, when deciding among alternative therapies, physicians treating patients with advanced lung carcinoma should give careful consideration to these problems and decide on the most suitable modality.

### *References*

[1] Hara, N., Ohta, M., Tanaka, K. *et al.* Assessment of the role of surgery for stage II bronchogenic carcinoma. *J. Surg. Oncol.* 1984; **25**: 153–158.

[2] Mountain, C.F. The biologic operability of stage III non-small cell lung cancer. *Ann. Thorc. Surg.* 1985; **40**: 60–64.

[3] McCaughan, J.S., Williams, T.E. and Bethel, B.H. Photodynamic therapy of endobronchial tumors. *Lasers Surg. Med.* 1986; **6**: 336–345.

[4] Hetzel, M.R. and Smith, S.G.T. Endoscopic palliation of tracheobronchial malignancies. *Thorax* 1991; **46**: 325–333.

[5] Balchum, O.J. and Doiron, D.R. Photoradiation therapy of endobronchial lung cancer: large obstructing tumors, nonobsrructing tumors, and early-stage bronchial cancer lesions. *Clin. Chest Med.* 1985; **6**: 255–275.

[6] McCaughan, J.S., Hawley, P.C. and Bethel, B.H. *et al.* Photodynamic therapy of endobronchial malignancies. *Cancer* 1988; **62**: 691–701.

[7] McChaughan, J.S. Jr., Hawley, P.C. and Walker, J. Management of endobronchial tumors: a comparative study. *Seminars in Surgical Oncology* 1998; **5**(1): 38–47.

[8] McChaughan, J.S. Jr., Barabash, R.D., Penn, G.M. and Glavan, B.J. Nd-YAG laser and photodynamic therapy for esophageal and endobronchial tumors under general and local anesthesia. Effects on arterial blood gas levels. *Chest* 1990; **98**(6): 1374–1378.

[9] Lam, S., Muller, N.L., Miller, R.R. *et al.* Predicting the response of obstructive endobronchial tumors to photodynamic therapy. *Cancer* 1986; **58**(10): 2298–2306.

[10] Sutedja, T., Baas, P., Stewart, F., van Zandwijk, N. A pilot study of photodynamic therapy in patients with inoperable non-small cell lung cancer. *European J. of Cancer* 1992; **23A**(8–9): 1370–1373.

[11] Spikes, J.D., Bommer, J.C. *et al.* Photo-bleaching of momo-L-asparartylechlorin e6 (NPe6) a candidate sensitizer for the photodynamic therapy of tumors. *Photochem. Photobiol.* 1993; **58**: 346–350.

[12] Sarvey, J.F., Monnier, P., Fontolliet, C. *et al.* Photodynamic therapy for early squamous cell carcinomas of the esophagus, bronchi, and mouth with m-tetra(hydroxyphenyl) chlorin. *Archives of Otolaryngology – Head & Neck Surgery* 1997; **123**(2): 162–168.

